# Verification of the Combimatrix influenza detection assay for the detection of influenza A subtype during the 2007–2008 influenza season in Toronto, Canada

**DOI:** 10.1186/1743-422X-6-37

**Published:** 2009-03-25

**Authors:** Shelly Bolotin, Ernesto Lombos, Rani Yeung, AliReza Eshaghi, Joanne Blair, Steven J Drews

**Affiliations:** 1 Ontario Agency for Health Protection and Promotion, 81 Resources Road, Toronto, Ontario, M9P 3T1, Canada; 2 Department of Microbiology, Mount Sinai Hospital, 600 University Avenue, Toronto, Ontario, M5G 1X5, Canada; 3 Department of Laboratory Medicine and Pathobiology, University of Toronto, 100 College Street, Room 110, Toronto, Ontario, M5G 1L5, Canada

## Abstract

The increase in adamantine resistance in influenza A (H3N2) and the emergence of oseltamivir resistance in influenza A (H1N1) has necessitated the use of rapid methodologies to detect influenza subtype. The purpose of this study was to evaluate the CombiMatrix influenza detection system compared to the FDA approved Luminex Respiratory virus panel (RVP) assay for influenza A subtyping. Verification of the CombiMatrix influenza detection system was carried out using the Luminex RVP assay as a reference method. A limit of detection (LOD) series was performed using the Luminex and CombiMatrix systems with both influenza A H3N2 and H1N1 viruses. Seventy-five clinical specimens were used in the study. Of these, 16 were influenza A (H3N2) positive and five were influenza A (H1N1) positive. Fifty-four specimens were influenza A negative or "no call" (inconclusive) or could not be subtyped. The LOD of the Luminex RVP assay was found to be 0.3 TCID_50_s/mL for influenza A (H3N2) and 16 TCID_50_s/mL for influenza A (H1N1). The LOD of the CombiMatrix influenza detection system was 200 TCID_50_s/mL for influenza A (H3N2) and 16 000 TCID_50_s/mL for influenza A (H1N1). The sensitivity of the CombiMatrix influenza detection system was 95.2% and the specificity was 100%. The CombiMatrix influenza detection system is an effective methodology for influenza A subtype analysis, specifically in laboratories with a constrained budget or limited molecular capabilities.

## Findings

Classification of seasonal influenza A into H3N2 or H1N1 subtypes is an important step in the characterization of circulating influenza A strains. The recent emergence of adamantine resistance in influenza A (H3N2) [1] and oseltamivir resistance in influenza A (H1N1) [2] has necessitated the use of methodologies that allow for rapid influenza sub-type analysis. A variety of both "home-brew" and commercial molecular assays that allow for sub-type analysis of influenza A subtypes are now available. Although many laboratories utilize "home-brew" subtyping methodologies [3,4], these are controversial due to multiple regulatory issues with the use of these assays leading to a growing movement for the use of commercial molecular diagnostics [5,6].

The purpose of this study was to evaluate the sensitivity and specificity of the CombiMatrix influenza A detection system for influenza A subtype analysis compared to the Luminex RVP assay, an FDA approved Respiratory Virus Panel (RVP) assay [7,8]. The CombiMatrix influenza A detection system is a commercial multiplex reverse transcriptase PCR (RT-PCR) assay and microarray detection system that can be used to identify hemagglutinin (HA) subtypes 1–16 and neuraminidase (NA) subtypes 1–9 [9]. Unlike conventional fluorescence-based microarrays, the CombiMatrix is an electrochemical system that detects current generated from redox enzymatic reactions (biotin-streptavidin) when DNA-probe hybridization occurs [10]. Verification of this technology was performed using specimens from Toronto, Canada collected during the 2007–2008 influenza season.

Nasopharyngeal specimens from patients from Toronto, Canada with influenza-like illness were sent to the Central Branch of the Ontario Public Health Laboratories (CPHL) during the 2007–2008 influenza season. Specimens were collected using the flocculated Starswab^® ^Multitrans Collection and Transport system (Starplex, Bolton, Canada).

Total nucleic acid was extracted from each specimen using the easyMag automated extraction system (bioMérieux, Montreal, Canada) as per the manufacturer's protocols. To control for extraction all specimens were tested for human target *gapdh *by using the *gapdh *RT-PCR kit (ABI, Foster City, CA) as per the manufacturer's instructions and as previously described [11].

Seventy-five specimens submitted to CPHL were included in this study (Table 1). Specimens were first screened by the Luminex RVP assay (Luminex Molecular Diagnostics, Toronto, Canada), a commercial FDA cleared assay that detects multiple respiratory pathogens including influenza A H3N2 and H1N1, influenza B, respiratory syncytial virus A (RSV A), respiratory syncytial virus B (RSV B), parainfluenza (PIV) 1, PIV2, PIV3, human rhinovirus A, human metapneumovirus (HMPV) and adenovirus. Nucleic acid from these specimens was then tested by the CombiMatrix influenza A detection system (CombiMatrix, Mukilteo, WA) as per the manufacturers instructions and as previously described [9]. RT-PCR reactions were carried out using the iCycler PCR thermocycler (Bio-Rad, Milpitas, CA). Influenza A subtyping steps were carried out using CombiMatrix influenza A detection arrays in conjunction with an Electrasense array reader as per the manufacturer's instructions and as previously described [9,10].

**Table 1:** Sensitivity and specificity of the CombiMatrix influenza detection system

	**Luminex positive**	**Luminex negative**
**CombiMatrix positive**	20	0
**CombiMatrix negative**	1	54
	Sensitivity = 95.2%	Specificity = 100%

The limit of detection (LOD) for both the Luminex RVP assay and the CombiMatrix influenza A detection system was determined using serial ten-fold dilutions of nucleic acid from influenza A/Brisbane/10/2007 (H3N2) virus from a clinical specimen and influenza A/PR/8/34 (H1N1) virus (Advanced Biotechnologies Inc., Columbia, MD) in PCR-grade water. The starting concentration of H3N2 virus was 10^6.8 ^TCID_50_s/mL and the starting concentration of H1N1 virus was 10^9.5 ^TCID_50_s/mL. The LOD was calculated using probit regression with a 95% confidence interval (95% CI) using SPSS 15 (SPSS Inc., Chicago, IL).

Of the 75 specimens characterized using the Luminex RVP assay (Table 1), 21 were influenza A positive and were subtyped by the Luminex RVP assay as either H3 (*n *= 16) or H1 (*n *= 5). Fifty-four specimens were defined by the Luminex RVP assay as both H3 and H1 "undetected". Of these, one specimen was detected by the Luminex RVP assay as influenza A but could not be typed and three specimens were detected as influenza A "No-call". The remaining 50 were identified as not influenza A by the Luminex RVP assay with the following distribution: no virus detected (17/50), influenza B (1/50), enterovirus/rhinovirus (7/50), HMPV (6/50), PIV1 (4/50), PIV2 (2/50), PIV3 (5/50), adenovirus (3/50), RSV A (2/50), mixed adenovirus and enterovirus/rhinovirus (1/50), mixed adenovirus and RSV A (1/50), mixed PIV2 and enterovirus/rhinovirus (1/50).

Compared to the Luminex RVP assay, the sensitivity of the CombiMatrix influenza detection system for the detection of an influenza A subtype (either H1 or H3) was 95.2% (Table 1). All 16 specimens characterized as H3 by the Luminex RVP assay were identified by the CombiMatrix influenza detection system. Four of five specimens characterized as H1 by the Luminex RVP assay were identified by the CombiMatrix influenza detection system. The specificity of the CombiMatrix influenza detection system for the detection of an influenza A subtype (either H1 or H3) was 100%.

The LOD of the Luminex RVP assay was found to be 0.3 TCID_50_s/mL for influenza A (H3N2) and 16 TCID_50_s/mL for influenza A (H1N1). The LOD of the CombiMatrix influenza detection system was 200 TCID_50_s/mL for influenza A (H3N2) and 16 000 TCID_50_s/mL for influenza A (H1N1).

Subtype analysis of influenza A is becoming increasingly prevalent in clinical microbiology laboratories, and is essential for public health surveillance of circulating strains, determination of annual vaccine mismatch and therapeutic decision making with regards to antiviral resistance.

Until recently, subtyping of influenza A was performed using antigenic determination of culture-grown virus. This method requires cell culture facilities and has a turn-around-time (TAT) of up to 10 days. The development of molecular methods for subtype determination has facilitated shorter TATs however the use of molecular technology is still hampered by the expertise required to perform the tests. This study evaluated the CombiMatrix influenza detection system for subtype analysis, a molecular detection system that is easier to operate than conventional real-time PCR instruments. This system was compared to the FDA-approved Luminex RVP assay.

While the CombiMatrix influenza detection system required less expertise to operate, its LOD was three logs lower than the Luminex RVP assay. The LOD for H1N1 subtypes was particularly low (16 000 TCID_50_s/mL) resulting in a negative result for one H1N1 isolate that was successfully subtyped using the Luminex RVP assay. This decreased sensitivity could be due to the inherent differences between the electrochemical CombiMatrix technology compared to the bead-based fluorescence technology of the Luminex platform, or as a result of differences in PCR efficiencies between the two systems. Despite the difference in LOD, the sensitivity of the CombiMatrix system was still high at 95.2%, making this instrument quite suitable as a secondary testing method.

In conclusion the CombiMatrix influenza detection system is an effective method for influenza A subtype analysis. Its ease of operation makes it suitable for laboratories with a limited budget or limited molecular knowledge.

## Competing interests

Funds for this project were provided by the Public Health Agency of Canada. The authors declare that they have no competing interests.

## Authors' contributions

EL carried out the Combimatrix and Luminex testing, RY and AE carried out the Combimatrix testing, JB assessed assay design, SB analyzed the data and wrote the manuscript, SJD conceived the idea for the study.
